# Neuroprotective Activity of *Hypericum perforatum* and Its Major Components

**DOI:** 10.3389/fpls.2016.01004

**Published:** 2016-07-11

**Authors:** Ana I. Oliveira, Cláudia Pinho, Bruno Sarmento, Alberto C. P. Dias

**Affiliations:** ^1^Nucleo de Investigação e Informação em Farmácia, Centro de Investigação em Saúde e Ambiente, Escola Superior de Tecnologia de Saúde do Porto – Instituto Politécnico do Porto, Vila Nova de GaiaPortugal; ^2^Agrobioplant Group (CITAB-UM), Department of Biology, University of Minho, BragaPortugal; ^3^Cooperativa de Ensino Superior Politécnico e Universitário, Instituto de Investigação e Formação Avançada em Ciências e Tecnologias da Saúde, Gandra PRDPortugal; ^4^Instituto de Investigação e Inovação em Saúde, PortoPortugal; ^5^Instituto de Engenharia Biomédica, PortoPortugal

**Keywords:** *Hypericum perforatum*, extracts, compounds, neuroprotection, antioxidant activity

## Abstract

*Hypericum perforatum* is a perennial plant, with worldwide distribution, commonly known as St. John’s wort. It has been used for centuries in traditional medicine for the treatment of several disorders, such as minor burns, anxiety, and mild to moderate depression. In the past years, its antidepressant properties have been extensively studied. Despite that, other *H*. *perforatum* biological activities, as its neuroprotective properties have also been evaluated. The present review aims to provide a comprehensive summary of the main biologically active compounds of *H. perforatum*, as for its chemistry, pharmacological activities, drug interactions and adverse reactions and gather scattered information about its neuroprotective abilities. As for this, it has been demonstrated that *H. perforatum* extracts and several of its major molecular components have the ability to protect against toxic insults, either directly, through neuroprotective mechanisms, or indirectly, through is antioxidant properties. *H. perforatum* has therefore the potential to become an effective neuroprotective therapeutic agent, despite further studies that need to be carried out.

## Introduction

*Hypericum perforatum*, also known as St. John’s wort, hypericum or millepertuis is a member of the family Hypericaceae and a herbaceous perennial plant native Europe, western Asia, and northern Africa. Nowadays it has a worldwide distribution. The crude drug, called herba hyperici, consists of the upper aerial parts of the plant collected just before or during the flowering period (Barnes et al., 2001; Greeson et al., 2001; Patocka, 2003).

*Hypericum perforatum* has been used as a medicinal plant for centuries, for the treatment of external and internal disorders. Externally, oily preparations of the plant may be applied to treat minor burns, wounds, skin inflammation, and nerve pain (Barnes et al., 2001; Greeson et al., 2001; Patocka, 2003). Internally, it is indicated for the treatment of anxiety and mild to moderately severe depression (Barnes et al., 2001; Greeson et al., 2001; Patocka, 2003; Butterweck and Schmidt, 2007) competing for status as a standard antidepressant therapy and being the only herbal alternative to synthetic antidepressants (Wurglics and Schubert-Zsilavecz, 2006).

*Hypericum perforatum* contains several classes of biologically active compounds. These constituents often vary in its concentration, due to genetic variation within the species and/or adulteration, ecological factors, time of harvesting, preparation and processing of sample material and storage conditions, such as exposure to light, time of harvesting. Important bioactive components are concentrated in buds, blossoms, and tips of twigs. Despite this variation, it is known that around 20% of the plant extract is comprised of bioactive compounds (Nahrstedt and Butterweck, 1997; Patocka, 2003; Wurglics and Schubert-Zsilavecz, 2006; Linde, 2009).

## Pharmacological Activity of *Hypericum perforatum*

St John’s wort most investigated pharmacological activity has been its antidepressant properties. There are several reports of higher *H. perforatum* effectiveness compared to placebo intake and a similar activity, when comparing to several antidepressant drugs. The exact mechanism of *H. perforatum*’s antidepressant activity is still unclear, as to which are the most relevant constituents. Early *in vitro* research suggested an antidepressant activity due to hypericin, through the inhibition of the monoamine oxidase (MAO) enzyme (Suzuki et al., 1984; De Vry et al., 1999; Gaster and Holroyd, 2000; Behnke et al., 2002; Linde et al., 2005; Linde, 2009; Rahimi et al., 2009). However, its concentration was too low to explain the clinical effects (Butterweck, 2003) detected. Further studies showed that hyperforin was capable of inhibiting the reuptake of serotonin, dopamine, noradrenaline, GABA, and L-glutamate (Chatterjee et al., 1998). Antidepressive activity was also reported in several flavonoids. Taking all these results in consideration and the fact that the mechanisms underlying depression are still not well understood, it is more likely that *H. perforatum*’s antidepressant activity is due to a multiplicity of bioactive compounds and not to a single constituent and/or mechanism of action (Greeson et al., 2001; Butterweck and Schmidt, 2007; Nahrstedt and Butterweck, 2010).

*Hypericum perforatum* also presents antimicrobial properties. Regarding the antibacterial activity, crude plant extracts of the aerial parts of the plant, fractions and isolated compounds have been tested, demonstrating positive results. Concerning specific compounds, hyperforin is reported to present antibacterial activity against *Staphylococcus aureus* and Gram-positive bacteria, such as *Streptococcus pyogenes* and *Corynebacterium diphtheriae* being considered the antibacterial agent of *H. perforatum*. As for antifungal properties the flavonoids quercitrin, hyperoside, avicularin, rutin, quercetin, and kaempferol were reported to present antifungal activity against *Helminthosporium sativum* (Schempp et al., 1999; Reichling et al., 2001; Saddiqe et al., 2010). Another compound extendedly studied, in what concerns its antimicrobial, more specifically, antiviral activity, is hypericin. This naphtodianthrone has demonstrated *in vitro* antiviral activity against a variety of viruses, light and oxygen influenced (Kubin et al., 2005). The *in vivo* studies did not, however, retrieve as promising results, possibly due to differences in terms of light irradiation (Karioti and Bilia, 2010) in many regions of the human body.

Besides its antiviral properties, hypericin has aroused interest in the scientific community for its antitumoral activity. Since hypericin is probably the most powerful photosensitizer found in nature and for its specific properties, such as a strong absorption at longer wavelength, minimal dark toxicity, certain tumor selectivity and much higher clearance rate from the host body than hematoporphyrins, its potential for antitumoral photodynamic therapy has been explored in several studies (Agostinis et al., 2002; Miskovsky, 2002; Martinez-Poveda et al., 2005b). Hyperforin and its stable, hydrosoluble derivate, aristoforin has also been reported to possess antitumoral activity, namely anticarcinogenic, antiproliferative, proapoptotic, antiinvasive, antimetastatic, and antiangiogenic effects (Hostanska et al., 2003; Schwarz et al., 2003; Dona et al., 2004; Martarelli et al., 2004; Gartner et al., 2005; Martinez-Poveda et al., 2005a; Rothley et al., 2009).

Anti-inflammatory, wound healing and anti-nociceptive effects have also been associated with *H. perforatum* (Motallebnejad et al., 2008; Suntar et al., 2010).

### *Hypericum perforatum*–Drug Interactions and Adverse Reactions

A number of clinically significant pharmacokinetic and pharmacodynamic interactions have been reported (Henderson et al., 2002) over the years suggesting that *H. perforatum* use concomitantly with several other drugs may represent its most relevant risk (Knuppel and Linde, 2004). These interactions are possibly due to a modulation of isoenzymes of the cytochrome P450 (CYP; Borrelli and Izzo, 2009), which metabolizes a series of pharmaceutical substances and an induction of P-glycoprotein, which is responsible for an increase of drugs’ excretion from the organism (Muller, 2003). **Table 1** summarizes the most known interactions.

**Table 1:** *Hypericum perforatum*–drug interactions.

Drug	Possible interaction	Type of interaction	Reference
Warfarin	Induction of CYP1A2 and CYP2C9	Pharmacokinetic	Karminsky and Zhang, 1997; Hammerness et al., 2003
Cyclosporin	Induction of CYP3A4 and P-glycoprotein transport	Pharmacokinetic	Henderson et al., 2002; Hammerness et al., 2003
	Risk of transplant rejection	Pharmacodynamic	Muller, 2003
Oral contraceptives	Induction CYP1A2 and CYP3A4	Pharmacokinetic	Ball et al., 1990; Schmider et al., 1997
	Inhibition of CYP2C9 and CYP2C19		Pfrunder et al., 2003
Theophylline	Induction of CYP1A2 and CYP2C9 and P-glycoprotein transport	Pharmacokinetic	Henderson et al., 2002; Hammerness et al., 2003; Wurglics and Schubert-Zsilavecz, 2006
Digoxin	Induction of CYP2C9, CYP2D6, CYP3A4, CYP1A2, CYP2C19, affecting P-glycoprotein transport, and reduction of drug’s plasmatic concentration	Pharmacokinetic	Barnes et al., 2001; Greeson et al., 2001; Henderson et al., 2002
HIV protease inhibitors	Induction of CYP3A4	Pharmacokinetic	Piscitelli et al., 2000
HIV non-nucleoside reverse transcriptase inhibitors	Induction of CYP3A4	Pharmacokinetic	Henderson et al., 2002; Hammerness et al., 2003
Anticonvulsivants	Induction of CYP2C9, CYP3A4, CYP1A2, and affecting P-glycoprotein transport	Pharmacokinetic	Barnes et al., 2001; Henderson et al., 2002
Phenprocoumon	Induction of CYP2C9, CYP2D6, CYP3A4, CYP1A2, CYP2C19, and affecting P-glycoprotein transport	Pharmacokinetic	Barnes et al., 2001
Nifedipin	Induction of CYP3A4;	Pharmacokinetic	Hammerness et al., 2003
	Induction of CYP3A4 and CYP2C19		Wang et al., 2007
Statins	Induction of CYP3A4;	Pharmacokinetic	Hammerness et al., 2003
	Induction of P-glycoprotein transport		Holtzman et al., 2006
Midazolam	Induction of CYP3A4;	Pharmacokinetic	Hammerness et al., 2003
Verapamil	Induction of first-pass CYP3A4 metabolism	Pharmacokinetic	Russo et al., 2013
Omeprazol, esomeprazole, and pantoprazole	Induction of CYP2C19	Pharmacokinetic	Wang et al., 2004
Loperamide	Theoretical induction of monoamine oxidase inhibitor-drug reaction	Pharmacokinetic	Khawaja et al., 1999
Ibuprofen	Increase of expression of glycoprotein G	Pharmacokinetic	Russo et al., 2013
Dexamethasone, prednisone, and budesonide	Induction of CYP3A4	Pharmacokinetic	Russo et al., 2013
Methadone and pethidine	Induction of CYP2D2	Pharmacokinetic	Dostalek et al., 2005
Dextromethorphan and oxicodone	Induction of CYP3A4	Pharmacokinetic	Nieminen et al., 2010
Voriconazole	Induction of CYP3A4, CYP2C19, and CYP2C9	Pharmacokinetic	Borrelli and Izzo, 2009
Erythromycin	Induction of CYP3A4	Pharmacokinetic	Borrelli and Izzo, 2009
Imatinib	Induction of CYP3A4 and P-gp	Pharmacokinetic	Smith et al., 2004
Triptans	Risk of increased serotoninergic effects with the possibility of an increased risk of adverse reactions	Pharmacodynamic	Barnes et al., 2001; Henderson et al., 2002; Hammerness et al., 2003
Selective serotonin reuptake inhibitors	Risk of increased serotoninergic effects with the possibility of an increased risk of adverse reactions	Pharmacodynamic	Barnes et al., 2001; Greeson et al., 2001; Henderson et al., 2002; Hammerness et al., 2003
Antineoplastic drugs directed against to topoisomerase II alpha	May antagonize therapeutic activity of the drugs	Pharmacodynamic	Hammerness et al., 2003
Thyroid agentes	Increase in thyroid-stimulating hormone	Pharmacodynamic	Ferko and Levine, 2001; Russo et al., 2013

As to what adverse reactions concerns, *H. perforatum* is referred to as generally well tolerated. When side effects occur they are considered mild and transient. The most common are gastrointestinal symptoms, dizziness, confusion, fatigue and/or sedation, skin reactions, restlessness or anxiety, headache, dry mouth and allergic reactions. These may occur in 1–3% of patients taking *H. perforatum*. There have also been described rare adverse reactions that include phototoxicity. Symptoms indicative of phototoxicity include dermal erythema, rash, and pruritus. These adverse reactions have been attributed to naphthodianthrones. Other rare adverse reactions described comprehend alopecia, neuropathy and mania (Barnes et al., 2001; Greeson et al., 2001; Hammerness et al., 2003; Schulz, 2006; Wurglics and Schubert-Zsilavecz, 2006; Russo et al., 2013).

## Neuroprotective Activity and *Hypericum perforatum*

The Central Nervous System (CNS) is known for being parti cularly sensitive to oxidative stress, which can be described as an imbalance between generation and elimination of reactive oxygen species (ROS) and reactive nitrogen species (RNS). This particular susceptibility of the brain is caused by a high metabolic rate, a low concentration of glutathione and antioxidant enzyme catalase (CAT) and a high proportion of polyunsaturated fatty acids. The general inability of neurons to divide explains some aging and neurodegenerative disease related loss of function, as neurons die, without chance to be replaced. Apoptosis and excitotoxicity are among the mechanisms that cause neuronal death, involving ROS and RNS Therefore oxidative stress plays an important role in the development of neurodegenerative diseases, such as Alzheimer’s and Parkinson’s disease and stroke. In order to prevent these cause-dependent diseases it is important to preserve redox environment and mitochondrial function of the cell. This can be achieved by avoiding the causes of oxidative stress and strengthen the defenses with the usage endogenous antioxidants and the intake of others. Endogenous antioxidants include enzymatic and non-enzymatic defenses. Enzymatic antioxidants react with reactive species and are, subsequently, efficiently recycled, preventing most of the formation of the toxic free radicals. Only small amounts of these enzymes are therefore needed to offer protection. Relevant enzymatic antioxidants are *Se*-glutathione peroxidase (GPx), CAT, and superoxide dismutase (SOD) that metabolizes superoxide, hydrogen peroxide (H_2_O_2_), and lipid peroxides. Non-enzymatic defenses can be divided in hydrophobic and hydrophilic antioxidants. Lipophilic antioxidants comprise α-tocoferol, carotenoids, and ubiquinone-10 and are mostly present in membranes and lipoproteins. Hydrophilic antioxidants include glutathione, histone-peptides, the iron-binding proteins transferring and ferritin, dihydrolipoic acid, melatonin, uric acid, and ascorbic acid. They can be found in cytosolic, mitochondrial, and nuclear aqueous compartments. These defense mechanisms are complementary to each other, due to the different species and cellular compartments that they act against. Exogenous antioxidants include several vitamins, such as A, E, and C, carotenoids and polyphenolic compounds, such as flavonoids (Pietta, 2000; Emerit et al., 2004; Silva et al., 2005; Zhao, 2005; Silva B.A. et al., 2008; Butterfield et al., 2007; Boots et al., 2008; Ansari et al., 2009).

Besides their usage in the prevention of neurodegenerative diseases, antioxidants could also be relevant on its treatment, as a single compound or in supplementary combination with drugs targeting other pathogenic mechanisms (Behl, 1999).

### Neuroprotective Activity of *Hypericum perforatum* Extracts

Lu et al. (2004) reported a neuroprotective effect of standard *H. perforatum* extracts on H_2_O_2_ trauma induced by an optimum concentration of 200 μM in rat pheochromocytome cell line PC12 (cell line widely used in *in vitro* model of neuronal injury and oxidative stress (Sasaki et al., 2003; Zou et al., 2010) within 24 h treatment. The extract improved the survival rate of neural cells, in a dose-dependent manner, at concentrations of 1 ~40 μg/mL, with a 133% improvement at 40 μg/mL. In extract concentrations ranging from 60 to 100 μg/mL, a decrease in cell viability was reported, maintaining, however, higher viability levels, when comparing to control (*p* < 0.05). *H. perforatum* extract, at concentrations of 1~100 μg/mL also decreased intra- and extracellular ROS levels, at 71 and 50%, respectively, when comparing to the control group. This is indicative of a limitation in the intracellular ROS generation during cell aerobic metabolism and of the extract’s entrance in the cells, with a consequent reduction of ROS levels. There also was reported a block in DNA fragmentation of H_2_O_2_-induced apoptosis (which reflects the endonuclease activity characteristic of apoptosis) at concentrations of 10 to 100 μg/mL (Lu et al., 2004; Zou et al., 2010). These results are in accordance with those described by Benedi et al. (2004) but with an insult of 300 μM H_2_O_2_. Pre-treatment with the standardized extract also attenuated caspase-3 activity, increased by H_2_O_2_ insult. Caspases, of which caspase-3 is the most widely studied, are a class of cysteine proteases, considered of extreme importance in the apoptotic process, triggering a proteolytic cleavage cascade in mammalian cells (Cohen, 1997).

Using the same biological model and insult, a flavonoid-rich extract, particularly in rutin, hyperoside, isoquercetin, avicularin, and quercitrin (Zou et al., 2004), proven significant protective effects against induced apoptosis, in the studied concentrations above 6.25 μg/mL (Zou et al., 2010). Similar results (regarding induced-apoptosis protection and attenuation of caspase 3-activity) have been described for a *H. perforatum*’s methanolic dried extract in human neuroblastoma cell line SK-N-MC. There was also observed, under the phase-contrast microscope, that cells treated with H_2_O_2_ (at a concentration of 10^-10^mM) for 5 h were detached from the dish, with cell rounding, cytoplasmic blebbing, and irregularity in shape. All of the morphological alterations described were less frequent among cells pre-treated with *H. perforatum*. The DAPI assay revealed nuclear condensation, DNA fragmentation, and perinuclear apoptotic bodies under the same treatment conditions with H_2_O_2_. The appearance of cells pre-treated with *H. perforatum* was, like described above, closer to that of control (Jang et al., 2002). *H. perforatum* standardized extract has also been described to have the ability to protect against enzymatic and non-enzymatic lipid peroxidation in rat brain, inhibiting NADPH-dependent lipid peroxidation and attenuating of non-enzymatic Fe^2+/^ascorbate-dependent lipid peroxidation in cerebral cortex mitochondria. Inhibition of lipid peroxidation was reported to be the results of the extract’s scavenging effect on NADPH and Fe^2+^/ascorbate generated free radicals (Benedi et al., 2004).

Glutamate, a neurotransmitter, which extracellular accumu lation leads to overstimulation of postsynaptic glutamate receptors, with consequent inhibition of intracellular glutathione synthesis and Ca^2+^ overload, can act as a toxic, leading to cell death (Ankarcrona et al., 1995; Breyer et al., 2007). Taking this in consideration Breyer et al. (2007) investigated the neuroprotective activity of *H. perforatum* extract in glutamate-induced cell death in hippocampal HT22 nerve cell line. Pre-incubation for 2–25 h with the extract and/or simultaneous incubation with glutamate and extract-incubation of the cells up to 8 h after glutamate exposition prevented cell death. There is no statistically significant difference between the control cells and the cells pre and co-incubated with the extract, whereas the glutamate-exposure cells decrease in cell viability more than 80%. It was also demonstrated that *H. perforatum* extract was able to counteract the energy losses induced by the glutamate. The authors concluded that the extract protected HT22 cells from glutamate-induced cytotoxicity by reducing or attenuating glutathione loss, calcium fluxes, energy status, and ROS-mediated cell death, but only up to 8 h after glutamate exposition.

Alzheimer’s disease is characterized by neuronal degeneration, particularly of pyramidal hippocampal neurons, entorhinal cortex, and other neocortical areas, which include the specific loss of cholinergic neurons in the median forebrain (Bains and Shaw, 1997). Besides that, two hallmarks of this neurodegenerative disorder are neurofibrillary tangles and senile plaques (Silva et al., 2004), the last mainly constituted by amyloid protein fibers, derived from an amyloid precursor protein, arranged in a so-called cross-β-pleated sheet conformation. This heterogeneous peptide in size is proven to be neurotoxic and this general toxicity appears mediated in some part by oxidative stress (Behl, 1999; Behl and Moosmann, 2002; Ansari et al., 2009). In fact, amyloid-β-induced toxicity on neuronal cells is proposed as a main route to neuronal loss in Alzheimer’s disease (Butterfield et al., 2007). Taking this in consideration and the protective characteristics of *H. perforatum* extracts, Silva et al. (2004) studied its potential neuroprotective action in β-amyloid-induced cell toxicity, through lipid peroxidation and Syto-13/PI assay. After incubating hippocampal wistar rat neurons with non-toxic concentrations of ethanolic extract *H. perforatum* and fractions, a significant inhibition of ascorbate/Fe^2+^-induced lipid peroxidation was observed on the fractions containing caffeoylquinic acids and flavonol glycosides, flavonol glycosides (quercetin-type), flavonol and biflavone aglycones and several phenols (19, 21 (*p* < 0.05), 77, and 98% (*p* < 0.001), respectively). *H. perforatum* extract and the fractions containing bianthraquinones, flavonol glycosides and flavonol and biflavone aglycones significantly inhibited lipid peroxidation, after cell incubation with β-amyloid_(25-35)_ 25 μM (*p* < 0.001), with levels lower than the control basal peroxidation. Cell viability was determined by Syto-13/PI assay. *H. perforatum* ethanolic extract and fractions containing flavonol glycosides, flavonol, and biflavone aglycones reduced cell death [65, 58, and 59%, respectively, when comparing to control (*p* < 0.001)]. Morphological analysis with cells stained with cresyl violet showed that after cell exposure to amyloid β-peptide a pattern of neuronal death was observed, specifically by a decrease in cell volume, nuclear condensation, appearance of apoptotic bodies and dendritic retraction. These alterations were not evident in the presence of *H. perforatum* extract and fractions containing bianthraquinones and flavonol glycosides. Accordingly with these results, the authors concluded that *H. perforatum* alcoholic extract and studied fractions have neuroprotective activity, which can be of relevance on preventing amyloid-β peptide neuronal degeneration. The senile plaques, in Alzheimer’s disease, are also enriched with reactive microglia and astrocytes (Akiyama et al., 2000). As immunocompetent cells of the brain, the microglia are able to counteract the deleterious effects of amyloid-β in Alzheimer’s disease. Taking this in consideration and amyloid β-peptide toxicity described above, Kraus et al. (2007) investigated the effects of the peptide on cell viability of microglia and a possible protective mode of action of *H. perforatum* extract by studying the influence of a pretreatment on cell survival. In BV2 and N11 cells (microglial cell lines) pretreated with *H. perforatum* ethanolic extract 50–100 μg/mL, the cell death evoked by treatment with amyloid-β_(25-35)_ and amyloid-β_(1-40)_ was significantly attenuated in a dose-dependent manner. It was concluded that treatment with *H. perforatum* ethanolic extract may restore or improve microglial viability, attenuating amyloid-β mediated toxicity in Alzheimer’s disease. Regarding biochemical and neurotransmitter alterations in the brain, this neurodegenerative disorder is characterized by a loss of cholinergic markers butyrylcholine and acetylcholine that are hydrolyzed butyrylcholinesterase and acetylcholinesterase, respectively. Cholinesterases are an ubiquitous class of serine hydrolases that hydrolyze choline esters with various efficiency (Talesa, 2001). The inhibitory effects of ethyl acetate, methanol and water *H. perforatum* extracts against butyrylcholinesterase and acetylcholinesterase were, therefore, investigated. Methanol extract was the one that, from the three tested, exerted the highest acetylcholinesterase inhibition (49.54 ± 4.44%), while the ethyl acetate extract had the best inhibition toward butyrylcholinesterase (50.79 ± 3.07%). The water extract presented no inhibitory effect in the tested concentrations (50–200 μg/mL). Another enzyme that has become a research target in neurodegenerative diseases investigation has been tyrosinase, a multifunctional enzyme, involved in neuromelanin production and damaged neurons associated with Parkinson’s disease. The three *H. perforatum* extracts described above were also tested against tyrosinase, and only the methanol extract was found to have a low inhibitory effect (19.21 ± 1.44%; Asanuma et al., 2003; Altun et al., 2013).

*Hypericum perforatum*’s neuroprotective activity has also been investigated in association with other compounds. For instance, a drug commonly used for the treatment of Parkinson’s disease is bromocriptine, which is reported to have strong free radical scavenging action *in vivo* and potent neuroprotective actions (Muralikrishnan and Mohanakumar, 1998; Mohanasundari et al., 2006). Due to several adverse effects of bromocriptine monotherapy, Mohanasundari et al. (2006) evaluated the combined effect of bromocriptine and *H. perforatum* alcoholic extract against 1-Methyl-4-phenyl-1,2,3,6-tetrahydropyridine (MPTP), a neurotoxin, in mice. Lipid peroxidation increased in the MPTP-treated group when compared to the control (in 91%). *H. perforatum* extract and bromocriptine alone lowered lipid peroxidation (*p* < 0.05) and the combined treatment showed even better results, when comparing with all the other groups (control, *H. perforatum* and MPTP-treated groups; *p* < 0.05). A mixture of *Panax quinquefolius*, *Ginkgo biloba*, and *H. perforatum* was proven to enhance retinal ganglion cell survival after axotomy, increasing the number of regenerating retinal ganglion cell in 87%, 21 days after optic nerve transaction. Effects of the herbal extracts mixture on the survival of axotomized retinal ganglion cells 7 days after axotomy showed a delay in cell death, offering, therefore, significant neuroprotection (*p* < 0.01) versus optical nerve transaction (Cheung et al., 2002). Axotomy-induced retinal ganglion cell death has been adopted as an animal model in the investigation of neuronal death in the CNS. It is known to be related to the activation of apoptotic pathways (Cheung et al., 2008), like caspase-3 and -9 (Cheung et al., 2004). When investigating the mechanisms underlying a neuroprotective activity of the herbal mixture described, Cheung et al. (2008) verified an apoptotic property, through the inhibition of cell’s nuclear fragmentation, with no effect on caspase-3 action, suggesting that the reduction of nuclear fragmentation was not achieved by the limitation of caspase-3 activation. The mixture, nonetheless, reduced the percentage of caspase-3-negative fragmented nuclei, though with no effect in caspase-3-dependent nuclear fragmentation, suggesting an inhibition by a caspase-3 independent pathway. Additionally, an intravitreal injection with wortannin, a phosphoinositide-3 kinase (PI3K) inhibitor, abolished the neuroprotective effect of the herbal mixture, indicating, according to the authors, that this effect was PI3K-dependent. It’s important to refer that PI3K–Akt (Akt: protein kinase identified in the AKT virus [also known as protein kinase B]) signaling pathway plays a critical role in mediating survival signals in a wide range of neuronal cell types (Brunet et al., 2001).

Since the removal of excessive ROS or suppression of their generation by antioxidants may be effective in the prevention of oxidative cell damage (Benedi et al., 2004), having therefore a neuroprotective effect, the protective antioxidant properties of *H. perforatum* will also be described.

El-Sherbiny et al. (2003) studied the effect of a dried ethanolic *H. perforatum* extract on brain oxidative status of naïve rats upon the administration of an amnesic dose of scopolamine. When in doses equivalent to the ones used to treat depression, the extract proven to inhibit brain malondialdehyde (MDA) formation, modulating also the activity of GPx and the glutathione levels without any alteration on any of the measured oxidative stress indices, suggesting that low doses of *H. perforatum* extract have antioxidant properties, protecting rat brain from elevated oxidative status due to the administration of antidepressants. These protective activities have also been reported in a hydroethanolic standardized and ethanolic (Benedi et al., 2004; Silva et al., 2005; Silva B.A. et al., 2008) extracts of *H. perforatum* through a direct radical scavenging activity on 1,1-diphenyl-2-pycryl-hydrazyl (DPPH) radical [EC_50_ = 109 μg/mL (Benedi et al., 2004), 49.3 ± 1.05 μg/dwb/mL (Silva B.A. et al., 2008), and 21 μg/dwb/mL (Silva et al., 2005)] and inhibition of xanthine oxidase (XO) activity (at a concentration of 5 μg/mL the reported inhibition of XO activity was by 16% with an IC_50_ = 68.3 μg/mL), which may also contribute to the scavenging action of the O^2-^ radical by *H. perforatum*. According to these findings, *H. perforatum* may have the ability to bind iron ions and a moderate to high direct scavenging action for hydroxyl radical, independent of any enzymatic activity (Benedi et al., 2004; Silva et al., 2005; Silva B.A. et al., 2008). Besides these properties, *H. perforatum* ethanolic and aqueous extracts has the ability to inhibit stress induced by 2,2′-azo-bis(2-methylpropianamidine) dihydrochloride (AAPH), an inductor of lipid peroxidation by formation of peroxyl radicals [IC_50_ = 50.4 ± 2.57 μg/dwb/mL (Silva B.A. et al., 2008) and 16.77 μmol of Trolox equivalent/g of fresh weight (Zheng and Wang, 2001)] and scavenge nitric oxide (NO) [through nitrite measurement, used as an estimate for the NO content (Cheung et al., 2008; Silva B.A. et al., 2008)] (10 μg/dwb/mL extract showed 78.7 ± 1.3% reduction of release of nitrite) and hypochlorous acid (through the reduction of 5-thio-2-nitrobenzoic acid (TNB; 50 μg/dwb/mL extract showed 17.1 ± 1.3% reduction in TNB oxidation; Silva B.A. et al., 2008).

As stated previously, Parkinson’s a neurodegenerative disease. In patients suffering from this pathology a reduction of mitochondrial complex I activity has been reported. Rotenone, a pesticide and specific inhibitor of this complex, causes tissue damage due to its toxic effect (Sanchez-Reus et al., 2007), with an involvement of oxidative damage (Sherer et al., 2003). Taken this in consideration, the potential antioxidant protective effect of a standardized extract of *H. perforatum* against rotenone-exposed rats was investigated. The extract was tested on brain MDA, GPx, CAT, MnSOD, and CuZnSOD expression and activities. Pretreatment of rats with *H. perforatum* extract before insult with rotenone resulted in an antioxidant activity with a decrease in brain MDA formation and an increase of SOD, CAT, and GPx levels. Regarding enzyme activities, *H. perforatum* extract recovered brain mRNA levels of SOD and GPx, altered by rotenone. In the case of CAT, pretreatment with the extract also revealed an antioxidant effect reducing mRNA levels of the enzyme elevated after rotenone administration, but this result was different from those of activity (Sanchez-Reus et al., 2007). This could be explained by an up-regulation of the relevant gene expression, protecting therefore the cell from rotenone toxicity (Alia et al., 2006).

Similarly to neuroprotective, also antioxidant properties of *H. perforatum* extracts have been tested in combined treatment regimens. When in simultaneous administration with bromocriptine and after an insult with MPTP, restoration of CAT, SOD, reduced glutathione (GSH), and GPx levels (CAT – 0.19 ± 0.01 to 0.57 ± 0.02; SOD – 1.97 ± 0.12 to 3.51 ± 0.08; GSH – 0.28 ± 0.02 to 0.53 ± 0.02; GPx – 8.6 ± 0.02 to 12.0 ± 0.07) to near normal (CAT – 0.58 ± 0.01; SOD – 3.83 ± 0.02; GSH – 0.62 ± 0.01; GPx –12.21 ± 0.17) was verified (Mohanasundari et al., 2006). The herbal mixture of *P. quinquefolius*, *G. biloba*, and *H. perforatum* described previously also exhibited anti-oxidant activity, mainly NO scavenging property, by lowering NO content in axotomized retinas [treatment with 30 mg of the mixture significantly lowered the amount of nitrite (*p* < 0.05) versus PBS-treated control group] without affecting NO synthase activity (Cheung et al., 2008).

### Neuroprotective Activity of *Hypericum perforatum* Major Compounds

*Hypericum perforatum*’s major compounds are described as neuroprotective in several studies, being this activity related with direct pathways, such as an *in vitro* and *in vivo* cytoprotective effect and indirect pathways, particularly through its antioxidant properties.

#### Quercetin

Quercetin, whose ability to interact with multiple cellular targets is likely the basis of its therapeutic activity (Dajas, 2012) has been described to protect against several insults, such as H_2_O_2_ (Dok-Go et al., 2003; Arredondo et al., 2004, 2010; Heo and Lee, 2004; Suematsu et al., 2011), linoleic acid hydroperoxide (LOOH; Sasaki et al., 2003), 6-hydroxydopamine (6-OHDA; Zhang et al., 2011), and *tert*-butylhydroperoxide (*t*-BOOH; Silva J.P. et al., 2008), in PC12 cells (Sasaki et al., 2003; Arredondo et al., 2004; Heo and Lee, 2004; Silva J.P. et al., 2008; Zhang et al., 2011), primary cultured rat cortical cells (Dok-Go et al., 2003) and rat cerebellar granule neurons (Arredondo et al., 2010) and human neuronal SH-SY5Y cells (Suematsu et al., 2011). In the first case, it’s described a cytoprotective action of quercetin 25 and 50 μM and 30–100 μM against H_2_O_2_ 200 and 400 μM (Arredondo et al., 2004; Heo and Lee, 2004), respectively, in PC12 cells. Similar effects were described, for primary cultured rat cortical cells, with quercetin at concentrations of 3 and 10 μg/mL, facing a H_2_O_2_-injury at 100 μM (IC_50_ = 4.1 μg/mL; Dok-Go et al., 2003). In primary rat cerebellar granule neurons a pretreatment with quercetin 25 μM significantly protected (*p* < 0.001) the cells from H_2_O_2_-injury at 60 μM. These findings were supported by morphological analysis of the cells (Arredondo et al., 2010). Heo and Lee (2004) also studied quercetin’s ability to block H_2_O_2_-induced membrane damage, verifying a significantly protective effect of this flavonol (*p* < 0.05). In human neuronal SH-SY5Y cells, quercetin increased the viability of H_2_O_2_-treated cells in a concentration dependent manner, becoming about 67% of that of the vehicle-treated ones at 100 μM. There were also suppressed H_2_O_2_-induced apoptotic features, such as DNA fragmentation, by co-treatment with quercetin, in a concentration dependent manner. In addition, quercetin suppressed the caspase cascade and pro-apoptotic Bax gene expression and increased anti-apoptotic Bcl-2 gene expression (Suematsu et al., 2011). Regarding LOOH insult, protective effects were reported in undifferentiated and differentiated PC12 cells with quercetin 25 and 50 μM, respectively, in pre- and co-incubation regimens (Sasaki et al., 2003). Pretreatment with quercetin 12.5–200 μM protected PC12 cells against 6-OHDA-induced damage (at a concentration of 1 mM) in a dose-dependent manner and significantly reduced (*p* < 0.05) the LDH leakage caused by the neurotoxin. Likewise, quercetin prevented 6-OHDA-induced cell apoptosis and attenuated 6-OHDA-induced NO over-production and iNOS over-expression. Morphological analysis of the cells treated with 6-OHDA revealed the presence of bright condensed dots, apoptotic bodies. There was also verified a reduction in colony density and cell size. Pretreatment with quercetin 25, 50, and 100 μM significantly attenuated nuclear condensation and at higher concentrations (50 and 100 μM) inhibited 6-OHDA-induced colony reduction and cell shrinkage (Zhang et al., 2011). Regarding NO production, it’s believed that its augment and consequent iNOS induction plays an important role in the initial phase of 6-OHDA-induced neuro-damage in *in vitro* and *in vivo* models (Lin et al., 2007). Taking this in consideration, Zhang et al. (2011) with a mechanistic study, tested the effect of quercetin on 6-OHDA-induced NO over-production and iNOS over-expression in PC12 cells, concluding that quercetin attenuated NO over-production via down-regulation of iNOS over-expression in 6-OHDA-treated cells. Silva J.P. et al. (2008) studied the protective effect of quercetin against *t*-BOOH-induced strand breaks. When added simultaneously with the insult, quercetin’s protective effect was significantly increased (*p* ≤ 0.001, compared with *t*-BOOH 200 μM), when comparing with a co-incubation regimen (*p* ≤ 0.01).

Likewise, pretreatment of HT22 cells with quercetin 5 and 10 μM significantly attenuated amyloid-β_(1-42)_-induced cytotoxicity (*p* < 0.001) and decreased 4-hydroxinonenal levels (an index of lipid peroxidation), in comparison to control (*p* < 0.001). Low doses of quercetin (5 and 10 μM) also mitigated morphological alterations induced by amyloid-β_(1-42)_, characterized by vacuolated soma and fragmented neurites, membrane blebbings and cell shrinkage, inhibiting, therefore amyloid-β_(1-42)_-induced apoptotic cell death (Ansari et al., 2009).

Liu et al. (2013) studied the effect of quercetin in the neurovascular unit (NVU) and its underlying mechanisms. The NVU comprises cerebral blood vessels and surrounding cells, such as astrocytes, neurons, and pericytes (Abbott, 2002) where the receptor for advanced glycation end products (RAGE) and low density lipoprotein receptor related protein-1 play an important role in the control of amyloid-β levels in the brain (Deane et al., 2009), maintaining it. After injecting amyloid-β_(25-35)_, quercetin, in concentrations from 5 to 40 mg/kg, was orally administrated, in male Kunming mice, for 8 days. Quercetin treatment improved the learning and memory capabilities and conferred neurovascular coupling protection, involving maintenance of the NVU integrity, reduction of neurovascular oxidation, modulation of microvascular function, improvement of cholinergic system, and regulation of neurovascular RAGE signaling pathway. The authors conclude on a possible quercetin mechanism through reduction of oxidative damage, inactivation of RAGE-mediated pathway and preservation of cholinergic neurons (Liu et al., 2013).

Silva B. et al. (2008) investigated the effects of *H. perforatum*’s phenolic compounds, such as quercetin, against neuronal excitotoxicity and mitochondrial disfunction. Quercetin 10 μM significantly reduced neuronal death due to kainate and NMDA-insult (*p* < 0.05). The authors correlated this protection with prevention of toxic-induced delayed calcium deregulation and the maintenance of mitochondrial electric potential. Quercetin 10 μM was also able to reduce mitochondrial lipid peroxidation and loss of mitochondrial transmembrane electric potential caused by oxidative stress ADP plus Fe-induced.

In Sprague-Dawley rats, with Parkinsonism induced by the neurotoxin 6-OHDA, a 14 days treatment with quercetin significantly increased the striatal dopamine (*p* < 0.05) by 34,56% and decreased the striatal protein carbonyl level (*p* < 0.05) by 49.69% compared with levels found in the 6-OHDA treated group (Haleagrahara et al., 2011). The increase of protein carbonyls is a proof of oxidatively damaged proteins, characteristic of Parkinson’s disease (McNaught et al., 2003). In zebrafish, considered a good model to study disorders of the dopaminergic system (Rink and Wullimann, 2001), co-treatment with quercetin (6 or 12 μM) significantly inhibited 6-OHDA-induced dopaminergic neuron loss (*p* < 0.05). However, there was no reversion of the neuron loss by quercetin after a 48 h exposure to 6-OHDA, what could be attributed to the poor permeability of quercetin across the blood–brain-barrier (Ossola et al., 2009; Zhang et al., 2011).

Regarding its antioxidant properties, quercetin has been shown to have an excellent *in vitro* antioxidant activity and it is considered the most potent scavenger of ROS, RNS, and peroxynitrite of the flavonoid family (Boots et al., 2008). In human hepatoma HepG_2_ cell line, quercetin 50 and 100 μM evoked a significant increase of intracellular GSH (76 ± 6 ng/mg/protein and 83 ± 7 ng/mg/protein, respectively), after 4 h treatment (*p* < 0.05; Alia et al., 2006), which can be expected, assuming the preparation of the cell against a potential oxidative insult (Myhrstad et al., 2002). This flavonol was also able to inhibit significantly ROS generation (*p* < 0.05), after insult with *t*-BOOH, therefore preventing or delaying conditions which favor oxidative stress in the cell (Alia et al., 2006). Increase of intracellular GSH has also been reported in rat primary cerebellar granule neurons, after a 24 h quercetin 25 μM pretreatment (146.3 ± 12%, in comparison to control; Arredondo et al., 2010). Regarding GSH synthesis, it’s important to refer the role of the Nuclear factor erythroid 2 related factor 2 (Nrf2)-dependent cytoprotective pathway in the induction of gene expression of enzymes involved in it. Taking into account that Nrf2 is a translocation factor, it is essential that it translocates to the nucleus in order to transactivate (Myhrstad et al., 2002; Zhang et al., 2013). Consequently, Liu et al. (2013) studied the activity of quercetin in nuclear translocation of Nrf2 in neurons. By the use of immunocytochemistry, there was verified that quercetin-treated cultures presented a major Nrf2 signal in both cytoplasm and, particularly, nucleus. The authors conclude that quercetin has the ability to cause nuclear translocation of Nrf2 in neuronal cultures, therefore activating the Nrf2 cytoprotective signaling pathway. Similarly, quercetin’s protective activity was verified by the inhibition of the oxidative injury induced by xanthine/XO in primary cultured rat cortical cells (quercetin 10 μg/mL and IC_50_ = 5.5 μg/mL; Dok-Go et al., 2003).

Quercetin prevents DNA single strand breakage and cytotoxicity, in U937 lymphoblast human cell line, caused by *t*-BOOH through its iron chelation properties. In fact, quercetin 100 μM and desferroxamine, an iron chelator (used as a control for this activity) prevented DNA cleavage generated by H_2_O_2_, whereas antioxidants trolox and *N*,*N*′-diphenyl-1,4-phenylenediamine were not efficient (Sestili et al., 1998). Metallic ions chelating is also involved in quercetin’s ability to diminish and prevent the oxidative hepatic damage produced by ethanol, besides interrupting the chain reaction that takes place on the lipid membrane. Pre-treatment with quercetin in mices treated with chronic doses of ethanol is more effective for CAT, selenium dependent-GPx, total GPx and GSH, which can be explained by a possible promotion of the antioxidant endogenous defenses (Molina et al., 2003).

According to Inal and Kahraman (2000), quercetin may be useful in reducing or preventing photobiologic damage, caused by ultraviolet A light, since it significantly decreases MDA levels (*p* < 0.05 and *p* < 0.001) and increases antioxidant defenses, namely SOD and CAT activities (*p* < 0.001), in Sprague-Dawley rats. In the same animal model, with Parkinsonism induced by 6-OHDA, a 14 days treatment with quercetin partially restored GSH levels, increasing it by 97.88% as compared with GSH in rats treated only with 6-OHDA (*p* < 0.05; Haleagrahara et al., 2011).

Antioxidant activities of quercetin have also been reported through its antiradical activity on DPPH [EC_50_ = 8.30 ± 1.03 μM (Silva B.A. et al., 2008), 11.34 ± 0.04 μM (Ramos et al., 2008) and 10.37 ± 1.53 μg/mL (Dok-Go et al., 2003)] and AAPH (EC_50_ = 29.4 ± 2.29 μM), lipid peroxidation inhibition potential (EC_50_ = 0.08 ± 1.90 μM; Silva B.A. et al., 2008) and inhibitory effect on NO synthase in a concentration-dependent manner, determined in rat cerebral homogenate and blood (IC_50_ = 63.06 and 57.54 μM, respectively; Luo et al., 2004).

Despite its beneficial activities, quercetin has also been reported to have toxic effects (Dok-Go et al., 2003; Arredondo et al., 2004, 2010; Boots et al., 2008; Ansari et al., 2009), being consequently important, to define, the therapeutical concentration of this compound (Ansari et al., 2009), for different pathologies, in order to maximize positive and minimize deleterious effects. It’s also relevant to refer that studies of *in vivo* quercetin’s neuroprotective activity remains controversial which can be due to the failure to examine prolonged exposures to micromolar levels of the flavonol and a required unattainable *in vivo* concentration (Ossola et al., 2009; Dajas, 2012).

#### Hyperoside

Hyperoside is the main active component of *H. perforatum* (Silva B.A. et al., 2008). Despite this, its neuroprotective activity has not been explored. Liu et al. (2005) evaluated hyperoside’s ability to prevent ROS generation and diminishing neuronal damage, in PC12 cells. Alone, hyperoside promoted the growth rate of the cells among the concentrations of 10–180 μg/mL, markedly between 10 and 120 μg/mL. As for its cytoprotective activity, hyperoside was effective preventing *t-*BOOH- and H_2_O_2_-induced toxicity, in a dose dependent manner, with the best improvement for 175% of control group at 160 μg/mL and 177% of control group at 100 μg/mL. These results were concordant to those of flow cytometry assay, where apoptotic cells formed via *t-*BOOH- and H_2_O_2_-induced toxicity were measured (sub-G1 peak). Hyperoside (160 and 100 μg/mL) attenuated cell death via apoptosis to 1.0 and 1.3%, respectively (Abbott, 2002). Protective properties of hyperoside in amyloid-β_(25-35)_-induced toxicity, in primary cortical rat neurons, were also studied. After exposure to amyloid-β_(25-35)_ (20 μM) for 24 h cell viability decreased to 63.1 ± 3.2%. Pretreatment for 30 min with hyperoside 5, 10, and 20 μM significantly increased cell viability [*p* < 0.05 for 5 and 10 μM and *p* < 0.01 for 20 μM, in comparison with amyloid-β_(25-35)_ treatment group]. Morphological analysis of the cells supported these results, with an effective reversion of amyloid-β_(25-35)_ neurite injury (observed as neurite loss and cleavage) after pretreatment with hyperoside (2.5, 5, 10, and 20 μM). Apoptosis was also reverted in a dose dependent manner, after pretreatment with hyperoside, by reversing the amyloid-β-induced mitochondrial disfunction, including mitochondrial membrane potential decrease, ROS production, and mitochondrial release of cytochrome *c*. Caspase-9 and caspase-3 activities were also significantly inhibited (*p* < 0.01), after pretreatment with hyperoside (5, 10, and 20 μM) and amyloid-β_(25-35)_-induced injury. Further study indicated that hyperoside can activate PI3K/Akt signaling, resulting in inhibition of Bad–Bcl_XL_ interaction, without intervening in Bad–Bcl-2 interaction. It was concluded that hyperoside can protect amyloid-β_(25-35)_-induced injury in primary cultured cortical neurons via PI3K/Akt/Bad/Bcl_XL_-regulated mitochondrial apoptotic pathway (Zeng et al., 2011). In the same biological model, the neuroprotective activity of hyperoside was investigated, by using an *in vitro* ischemic model of oxygen-glucose deprivation followed by reperfusion (OGD-R). Pretreatment with hyperoside (3, 10, 30, and 100 μM) for 24 h was able to significantly protect cultured cortical neurons from OGD-R injury (*p* < 0.05 for 3 μM and *p* < 0.01 for 10, 30, and 100 μM, in comparison to OGD-R group). In order to investigate the protective effect of hyperoside in neuronal excitotoxicity that can occur after OGD-R injury, cultured cortical neurons were exposed to glutamate 200 μM combined with glycine 10 μM for 2 h, followed by 24 h reperfusion. Pretreatment with hyperoside 24 h prior to the glutamate-exposure reversed the degradation of cell viability in a concentration dependent manner and significantly increased the neuronal survival rate (*p* < 0.05 for 1 μM and *p* < 0.01 for 3, 10, 30, and 100 μM in comparison to glutamate-exposure group). Hyperoside (10 μM) also relieved NMDA receptor-induced [Ca^2+^]_i_ elevation (*p* < 0.01, compared to NMDA group). NMDA receptor mediates Ca^2+^ influx, which is responsible for excitotoxicity (Liu et al., 2012). As for possible related mechanisms, the authors describe attenuation of CaMKII phosphorylation caused by OGD-R lesions. Hyperoside also lessened iNOS expression induced by OGD-R via inhibition of Nf-kB activation and ameliorated extracellular signal-regulated kinase, c-Jun NH_2_-terminal kinase, and Bcl-2 family-related apoptotic signaling pathways (Liu et al., 2012), related to NO signaling pathway.

As for its antioxidant activity, hyperoside is known for its ROS scavenging activity (Liu et al., 2005). It has active antiradical activity on DPPH (EC_50_ = 6.38 ± 1.06 μM) and APPH (EC_50_ = 11.5 ± 1.76 μM), lipid peroxidation inhibition potential (EC_50_ = 5.37 ± 1.05 μM; Silva B.A. et al., 2008) and inhibitory effect on NO synthase in a concentration-dependent manner, determined in rat cerebral homogenate and blood (IC_50_ = 56.23 and 158.49 μM, respectively; Luo et al., 2004).

#### Quercitrin

Quercitrin is thought to possibly overcome quercetin in its antioxidant and neuroprotective activity due to its high bioavailability in the digestive track (Hollman et al., 1995). Despite this, few studies have been published focusing on the neuroprotective activity of quercitrin. Rattanajarasroj and Unchern (2010) studied the neuroprotective effects of quercitrin on amyloid-β_(25-35)_-induced injury in cultured hippocampal rat neurons, as well as its possible mechanisms. Co-incubation of quercitrin (50 and 100 μM) and amyloid-β_(25-35)_ for 72 h significantly increased cell viability (*p* < 0.01), in comparison to cells only subjected to amyloid-β_(25-35)_ injury. These results were supported by the analysis of cell death, through LDH leakage assay. Here and in all experimental conditions, the magnitude of cell death was correlated with the percentage of cell viability in a complementary manner. Neuroprotective potential of quercitrin, using a rat primary-isolated retinal ganglion cells cultured under three types of stress conditions: hypoxia, excessive glutamate levels, and oxidative stress, was also evaluated. After 12 h of hypoxia stress, the retinal ganglion cells survival rate was reduced in cells without quercitrin to 55.5 ± 10%. Treatment with quercitrin 100 nM and 1 μM significantly increased retinal ganglion cell viability (*p* < 0.05; Nakayama et al., 2011). Regarding glutamate-induced cell death similar results were verified, with the quercitrin-treated retinal ganglion cells [increase in cell viability, in the same concentrations referred above (*p* < 0.05)].

As for its antioxidant activity, co-exposure of quercitrin (50 and 100 μM) and amyloid-β_(25-35)_ for 72 h significantly decreased cellular lipid peroxidation in a concentration dependent manner (*p* < 0.05, compared to amyloid-β_(25-35)_-exposure group). There were, however, no significant effects on amyloid-β_(25-35)_-induced ROS accumulation. It was hypothesized that a long exposure of cells to quercitrin could alter intracellular antioxidant defense system, including the production of GSH, SOD, and GPx. However, only GPx significantly increased its activity after co-exposure to quercitrin and amyloid-β_(25-35)_ (*p* < 0.05; Rattanajarasroj and Unchern, 2010).

Antioxidant activities of quercitrin have also been reported through its antiradical activity on DPPH (EC_50_ = 13.0 ± 1.10 μM) and lipid peroxidation inhibition potential (Wagner et al., 2006; Silva B.A. et al., 2008; EC_50_ = 7.33 ± 1.16 μM; Silva B.A. et al., 2008).

#### Rutin

Neuroprotective properties of rutin, a flavonoid with a wide range of biological activities, have been investigated. Wang et al. (2012) studied the interference of rutin in the pathogenic factors of Alzheimer’s disease. Morphological analysis of amyloid-β fibrillization revealed that amyloid-β_42_ co-incubation with rutin 200 μM inhibited by more than 95% fibril formation. In SH-SY5Y cells rutin 20 μM significantly inhibited amyloid-β_42_ cytotoxicity (*p* < 0.05), also restoring cells fluorescent intensity ratio value in a concentration-dependent manner, which is, according to the authors, indicative of an attenuation of amyloid-β_42_-induced mitochondrial dysfunction. In order to determine rutin’s ability to protect against an oxidative damage, SH-SY5Y cells were treated with amyloid-β_42_, in the presence and absence of rutin. The flavonoid decreased amyloid-β_42_-induced ROS production in a concentration dependent-manner, with a significant inhibition when rutin 8 μM was employed (*p* < 0.05). Likewise, MDA levels were significantly decreased with rutin 0.8 and 8 μM (*p* < 0.05). Regarding the regulatory effect of rutin on GSH content, rutin 0.8 and 8 μM increased, in a concentration-dependent manner, GSH content of BV-2 microglial cells and decreased GSSG levels. The GSH/GSSG ratios were also decreased by amyloid-β_42_ and increased with the addition of rutin 8 μM (*p* < 0.05). Rutin’s effects on aldehyde dehydrogenase 2 (enzyme which metabolizes acetaldehyde into non-toxic acetate; ALDH2) activity in HT22 cells were also studied. Pretreatment with rutin 1 μg/mL, significantly inhibited ethanol-induced cell death (*p* < 0.01). Co-treatment with rutin also significantly reversed ethanol-increased Bax (*p* < 0.05, compared with ethanol), caspase 3 activity, and decreased Bcl-2 and Bcl-xL protein expression (*p* < 0.01, compared with ethanol). In order to clarify the mechanisms involved in rutin’s protective effects, an ALDH2 inhibitor, daidizin, was employed. Taking into account that pretreatment with rutin also lowered cytochrome *c* expression (involved in ethanol-induced apoptosis in HT22 cells; *p* < 0.01) and increased ALDH2 expression, it is concluded that rutin protects HT22 cells against ethanol-induced neurotoxicity by increasing ALDH2 activity (Song et al., 2014). Nakayama et al. also investigated the effects of rutin under the three types of stress conditions mentioned above. Regarding hypoxia stress, the retinal ganglion cells survival rate was reduced in cells without rutin 56.0 ± 3.1%. Treatment with rutin 1, 10, and 100 nM significantly increased retinal ganglion cell viability (*p* < 0.05). As for glutamate-induced cell death similar results were verified [increase in cell viability, in the same concentrations referred above (*p* < 0.05)]. Under oxidative stress conditions, the activity of caspase-3 and calpain were studied, in order to investigate the effect of rutin in apoptotic and necrotic cell death signaling, respectively. Calpains are a family of cytosolic cysteine proteinases whose enzymatic activities depend on Ca^2+^ and are believed to function in various biological processes, including cell death, more specifically, necrosis. Rutin at final concentrations of 1, 10, and 100 nM showed significant reduction of caspase-3 activity under hypoxia and by glutamate stress (*p* < 0.05) and of calpain activity by oxidative stress (*p* < 0.05; Goll et al., 2003; Nakayama et al., 2011).

Khan et al. (2009) investigated the protective effects of rutin on cerebral ischemia on Wistar rats. Pretreatment with rutin (25 mg/kg, for 21 days) protected the animals from motor deficit and lead to recovered motor coordination (*p* < 0.05, in comparison with the lesion group), improving, therefore, the neurological outcomes. It was also verified a significant attenuation (*p* < 0.05) on thiobarbituric acid reactive species, H_2_O_2_ levels and protein carbonyl content, in comparison to lesioned rats. Morphological analysis of rats’ brains revealed that activation of p53 up-regulation (associated with neuronal cell death in cerebral ischemia) was also attenuated by rutin. These results lead the authors to conclude that rutin offered significant protection on middle cerebral artery occlusion rats, probably due to inhibition of neurological deficit, lipid peroxidation, p53 expression and increase in endogenous antioxidant defense enzymes (data shown downward), evoking neuroprotection to the degenerating dopaminergic neurons (Khan et al., 2012). Taking the previous results into consideration, Khan et al. (2012) investigated the neuroprotective activity of rutin on 6-OHDA-induced Parkinson’s disease in rats. Similar results were obtained concerning the rutin’s protective activity, leading the authors to suggest that the consumption of rutin may have positive effects on the prevention on neurological disorders, such as Parkinson’s disease. Regarding Alzheimer’s disease and in following the study of Wang et al. (2012) and Xu et al. (2014) investigated the effects of rutin on APPswe/PS1dE9 transgenic mice. These animals overproduce human amyloid-β_40_ and amyloid-β_42_, also developing progressive cerebral β-amyloid deposit and learning and memory impairment, being considered animal models for Alzheimer’s disease (Garcia-Alloza et al., 2006). Oral administration of rutin (100 mg/kg for 6 weeks) significantly attenuated memory deficits, associated with the reduction in β-amyloid oligomer formation. It also improved spatial memory (*p* < 0.05, compared with disease’s control group) in transgenic mice. Similarly to *in vitro* studied performed by Wang et al. (2012), there was also reported a protective activity by rutin in the attenuation on β-amyloid-induced oxidative stress and lipid peroxidation. Rutin was also able to reduce neuro-inflammation in transgenic mice by attenuating microgliosis and astrocytosis (Xu et al., 2014).

Rutin is considered to have powerful antioxidant capacity against several antioxidant *in vitro* systems, being a concentration dependent property (Yang et al., 2008). Alike quercetin, in HepG_2_ cells, rutin 100 μM significantly increased intracellular GSH (52 ± 2 ng/mg/protein; *p* < 0.05). Despite its similar effects, rutin demonstrated to be less active than quercetin, which could be related to its lower bioavailability (Alia et al., 2006).

As to *in vivo* studies, antioxidant activities have also been reported (La Casa et al., 2000; Kamalakkannan and Stanely Mainzen Prince, 2006; Khan et al., 2009; Xu et al., 2014). Pre-treating Wistar rats with rutin (25 mg/kg, for 21 days) significantly restored (*p* < 0.05) GSH, GPx, GR, SOD, and CAT in hippocampus (GPx – from -35.69 to 31.31%; GR – from -45.12 to 48.65%; SOD – from -45.18 to 39.42%; CAT – from -54.28 to 66.96%) and frontal cortex (GPx – from -41.53 to 45.56%; GR – from -46.36 to 36.53%; SOD – from -50.51 to 68.73%; CAT – from -55.95 to 43.80%), in comparison to lesioned rats (Khan et al., 2009).

Antioxidant activities of rutin have also been reported through its antiradical activity on DPPH [Ramos et al., 2008; Silva B.A. et al., 2008; Yang et al., 2008; EC_50_ = 11.3 ± 1.06 μM (Silva B.A. et al., 2008) IC_50_ = 18.27 ± 0.62 μM (Ramos et al., 2008)], AAPH [EC_50_ = 31.5 ± 4.93 μM (Silva B.A. et al., 2008)], lipid peroxidation inhibition potential (EC_50_ = 8.98 ± 1.03 μM) and scavenging of superoxide radicals (IC_50_ = 0,13 mg/mL) and hydrogen peroxide (IC_50_ = 24 μg/mL; Yang et al., 2008).

#### Hypericin

The effect of hypericin (neuroprotection or apoptosis) on the transcription factor NF-kB, which is involved in regulation of genes relevant in several cellular processes, like neuronal survival and inflammatory response, has been accessed (Kaltschmidt et al., 2002). One hour treatment of cerebellar granule cells with 0.1 μM hypericin resulted in activation of NF-kB, which is further enhanced with 1 or 10 μM hypericin. Despite of the hypothesis that long-lasting activation of the transcription factor would result in neuroprotection, a 24 h treatment with different hypericin concentrations lead to a loss of the NF-kB activation previously observed. Basing on these results the authors investigated the effect of hypericin in Fe^2+^ ion-induced cell death. While low concentrations of hypericin (0.1 and 1 μM) had no effect on cell survival, 10 μM exerted cell death up to 100% after 24 h treatment, having therefore a synergistic effect with Fe^2+^. It was concluded that stimulus like hypericin, depending on the gene promoters that are activated, may have a neuroprotective therefore anti- or apoptotic activity (Kaltschmidt et al., 2002).

Low antioxidant activity of hypericin have been reported through its lipid peroxidation inhibition potential (EC_50_ = 21.0 ± 2.86 μM; Silva B.A. et al., 2008).

#### Kaempferol

Filomeni et al. (2012) studied the ability of kaempferol to protect SH-SY5Y cells and primary neurons from rotenone-induced toxicity. Pre-treating cells with kaempferol 30 μM for 1 h prior to a 1 μM rotenone-insult significantly counteracted rotenone-induced toxicity (*p* < 0.01). Microscopic morphologic analysis indicated that kaempferol was able to inhibit rotenone-induced round shape phenotype and cell detachment, characteristics of apoptotic process. At a molecular level, kaempferol was proven to significantly inhibit rotenone-induced caspase-3 and -9 cleavage (*p* < 0.001). Kaempferol was also able to preserve and restore mitochondrial function upon rotenone-mediated challenge, at least if provided before caspases activation, within upon 12 h. Underlying kaempferol protective activity was its autophagic’s ability, prior demonstrated in carcinoma cells by altering cellular energetics. The authors conclude that this maintained at lower doses and protects neuronal cells against from rotenone-induced toxicity. Note that autophagy has been observed to be deregulated in Parkinson’s disease (Chu et al., 2009; Filomeni et al., 2010, 2012).

Kaempferol was also investigated for its ability to protect neurons from excitotoxicity and mitochondrial dysfunction. Results were similar to those of quercetin, what lead the authors to conclude for a possible neuroprotective activity induced by the antioxidant properties of these compounds (Silva B. et al., 2008).

Antioxidant activities of kaempferol have been reported through its antiradical activity on DPPH (EC_50_ = 21.3 ± 1.04 μM) and lipid peroxidation inhibition potential (EC_50_ = 0.69 ± 1.62 μM; Silva B.A. et al., 2008).

#### Biapigenin

Biapigenin is a sparingly studied compound for its possible neuroprotective activity. It was studied (as well as quercetin and kaempferol) its ability to protect neurons from excitotoxicity and mitochondrial dysfunction. Besides the results already described, that were similar for biapigenin, this biflavone significantly affects mitochondrial bioenergetics (*p* < 0.001, in comparison to insult) and decreased the mitochondria’s ability to accumulate calcium (*p* < 0.05, in comparison to control; Silva B. et al., 2008). Biapigenin modulates the mitochondrial permeability transition pore, reducing calcium burden and contributing against excitotoxic insults (Silva et al., 2010).

Antioxidant activities of biapigenin have been reported through its lipid peroxidation inhibition potential (EC_50_ = 5.10 ± 1.11 μM; Silva B.A. et al., 2008).

#### Hyperforin

Hyperforin is the major lipophilic constituent of *H. perforatum* (Albert et al., 2002) and considered the major active compound for the anti-depressant activity (Singer et al., 1999).

Dinamarca et al. (2008) studied the ability of a hyperforin synthetic analog, tetrahydroperforin (IDN 5706) on the reduction of β-amyloid deposition and the improvement of spatial learning acquisition, destabilizing amyloid_β_-acetylcholinesterase interaction. It is important to refer that *in vivo* and *in vitro* studies are indicative of an enhancement of amyloid_β_ aggregation and amyloid fibril formation by acetylcholinesterase (Dinamarca et al., 2008). This study showed that IDN 5706 decreased the formation of β-amyloid fibrils *in vitro*, depolymerizing them.

An oral administration of 1.25 mg/kg/day, for 7 days, in rats, improved learning ability from the second day onward. Additionally the memory of the learned responses acquired during the administration time and training was retained after 9 days without further treatment or training. Klusa et al. (2001) also verified that on mice, a single dose of 1.25 mg/kg of hyperforin improved memory acquisition and consolidation and completely reversed scopolamine-induced amnesia. The authors concluded that hyperforin possesses memory enhancing properties, being potentially considered as an antidementia compound (Klusa et al., 2001).

*In vivo* and in transgenic mice, Dinamarca et al. (2008) verified that IDN 5706 was able to remove the acetylcholinesterase present in the plaques and improved the animal behavior, indicating that the presence of the enzyme was correlated to the behavioral impairment observed.

In a subsequent work, Inestrosa et al. (2011) studied the *in vivo* effects of IDN 5706 on β-amyloid neurotoxicity using young transgenic mice. Five months old mice were treated for 10 weeks, tested for spatial memory and their brains analyzed through several techniques. The authors reported that IDN 5706 significantly reduced spatial memory impairments, tau hyperphosphorylation, β-amyloid oligomer accumulation and increased long-term potentiation, suggesting that this compounds could be a novel pharmacological tool for the treatment of Alzheimer’s disease (Inestrosa et al., 2011).

## Conclusion

*Hypericum perforatum* has been used in traditional medicine for several hundred years. Despite of not fully studied or understood, the extract and isolated compounds of this plant have demonstrated neuroprotective activities. Neuroprotection can be achieved by a direct action on one or several mechanisms, such as an anti-apoptotic effect, or indirectly, through antioxidant properties. Chemically, structure-activity relationships suggest that sugar side chain of flavonoids might be important for neuroprotective activities (Nakayama et al., 2011) and multiple hydroxyl groups confer these compounds substantial antioxidant properties (Heim et al., 2002). Taken together, the data collected suggests a protective effect of *H. perforatum* and some of its major compounds in neurotoxicity, thus a possible beneficial activity in neurodegenerative disorders, such as Alzheimer’s and Parkinson’s disease. Nonetheless, further studies are needed to fully understand and characterize the activity of this plant and its compounds and its possible therapeutic activity.

## Author Contributions

All authors contributed on the conception and design of the work. AO and CP specifically intervened on the acquisition and interpretation of data and AO, BS, and AD collaborated on the structure of the work. AO was the main responsible for drafting. CP, BS, and AD critically revised it. All authors approved the final version of this work and agree to be accountable for all aspects of the work.

## Conflict of Interest Statement

The authors declare that the research was conducted in the absence of any commercial or financial relationships that could be construed as a potential conflict of interest.
